# Macrophages attenuate the transcription of *CYP1A1* in breast tumor cells and enhance their proliferation

**DOI:** 10.1371/journal.pone.0209694

**Published:** 2019-01-07

**Authors:** Sofia Winslow, Anica Scholz, Peter Rappl, Thilo F. Brauß, Christina Mertens, Michaela Jung, Andreas Weigert, Bernhard Brüne, Tobias Schmid

**Affiliations:** Institute of Biochemistry I, Faculty of Medicine, Goethe-University Frankfurt, Frankfurt, Germany; University of South Alabama Mitchell Cancer Institute, UNITED STATES

## Abstract

While aberrant cells are routinely recognized and removed by immune cells, tumors eventually escape innate immune responses. Infiltrating immune cells are even corrupted by the tumor to acquire a tumor-supporting phenotype. In line, tumor-associated macrophages are well-characterized to promote tumor progression and high levels of tumor-infiltrating macrophages are a poor prognostic marker in breast cancer. Here, we aimed to further decipher the influence of macrophages on breast tumor cells and determined global gene expression changes in three-dimensional tumor spheroids upon infiltration of macrophages. While various tumor-associated mRNAs were upregulated, expression of the cytochrome P450 family member *CYP1A1* was markedly attenuated. Repression of *CYP1A1* in tumor cells was elicited by a macrophage-shaped tumor microenvironment rather than by direct tumor cell-macrophage contacts. In line with changes in RNA expression profiles, macrophages enhanced proliferation of the tumor cells. Enhanced proliferation and macrophage presence further correlated with reduced *CYP1A1* expression in patient tumors when compared with normal tissue. These findings are of interest in the context of combinatory therapeutic approaches involving cytotoxic and immune-modulatory compounds.

## Introduction

Tumors shape their local microenvironment, which is formed by diverse stromal cells [1, 2]. An important component of the tumor microenvironment are immune cells, which infiltrate the tumor to exert both anti- and pro-tumoral functions. Macrophages (MΦ) are amongst the most abundant infiltrating leukocytes in many tumor types [3]. Their infiltration has been linked to poor outcome *e*.*g*. in breast cancer [4]. While MΦs have been shown to influence tumor promoting processes such as angiogenesis and migration [5, 6], the consequences of the interaction between tumor cells and MΦs on gene expression in tumor cells have not been comprehensively investigated so far.

Cytochrome P450 enzymes (CYPs) are crucial to detoxify harmful substances, such as polycyclic aromatic hydrocarbons (PAH), and a number of CYPs are induced by exposure to their potential substrates, *i*.*e*. xenobiotics [7]. Yet, the metabolizing activity of CYPs can produce even more potent carcinogens by the formation of reactive intermediates [8]. Along these lines, CYP1A1 expression was recently shown to be important for the DNA-damaging activity of 3-methylcholanthrene (MCA). Furthermore, reduced CYP1A1 expression in MΦs attenuated tumor formation in the MCA-induced fibrosarcoma model [9]. In line, polymorphisms associated with higher CYP1A1 activity have been associated with elevated breast cancer risk [10, 11]. Interestingly, CYP1A1 expression commonly appears to be repressed by inflammatory mediators [12, 13].

We aimed to investigate the effect of MΦs on gene expression in MCF7 breast tumor cells. *CYP1A1* mRNA expression was down-regulated in tumor cells upon exposure to MΦ-derived factors in a contact-independent manner. In parallel, MΦs increased proliferation of tumor cells. High MΦ numbers and reduced *CYP1A1* expression was further seen in human tumors, when compared to normal tissue.

## Results

### Impact of MФ infiltration on gene expression in three-dimensional breast tumor spheroids

**Fig 1:**
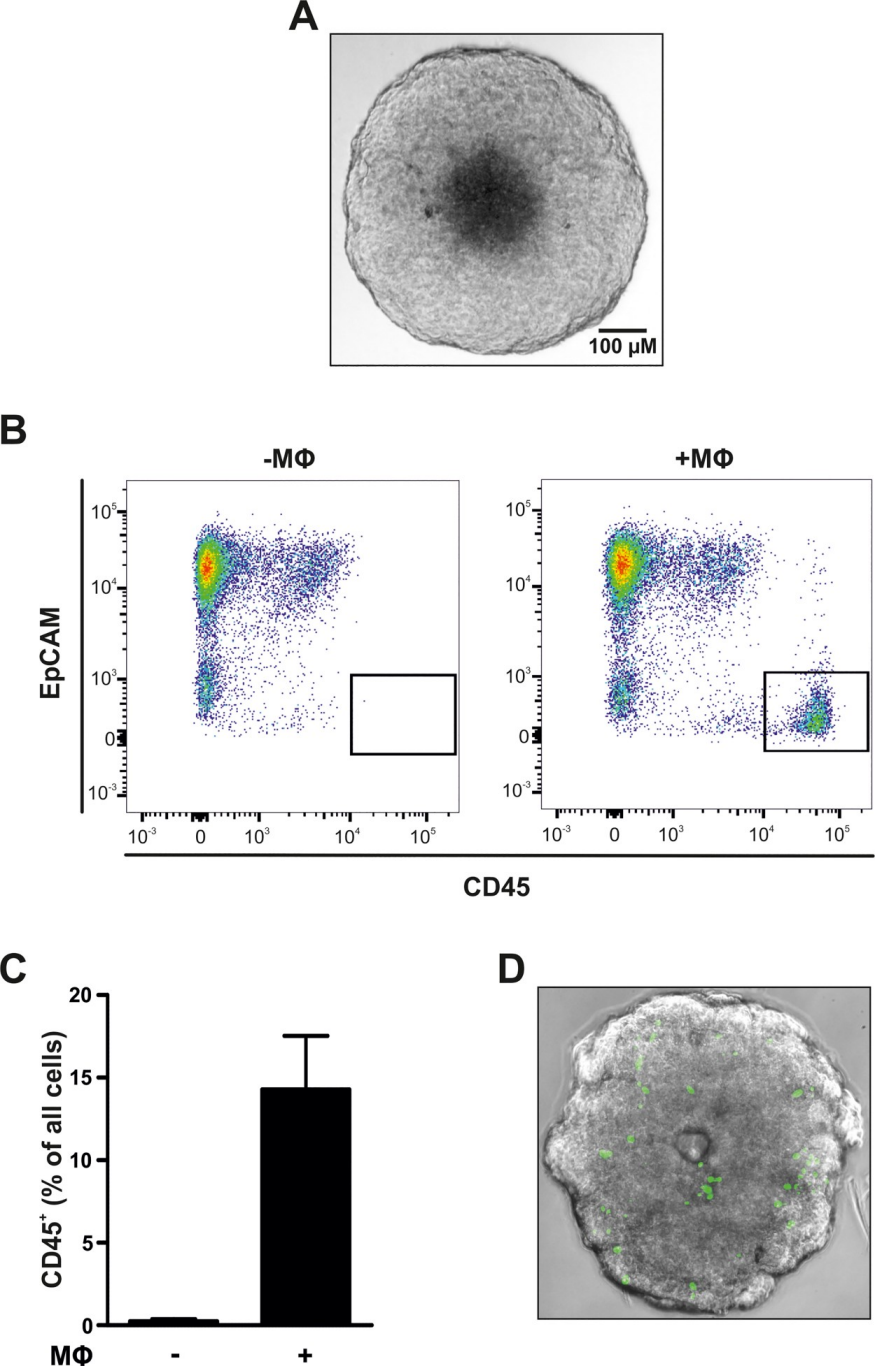
Macrophage infiltration into tumor spheroids. (A) 7.5 x 10^3^ MCF7 cells were seeded in agarose-coated 96-well plates to form three-dimensional spheroids. Picture is representative for 5 days old MCF7 tumor spheroids. (B) 7.5 x 10^4^ CD14^+^ cells were added to 5 days old spheroids. Cellular composition of the spheroids subsequently cultured for 2 days in the absence (*left panel*) or presence (*right panel*) of CD14^+^ cells was determined by FACS analysis of EpCAM^+^ tumor cells and CD45^+^ immune cells. Graphs are representative for 3 independent experiments. (C) MΦ infiltration was determined as the proportion of CD45^+^ cells relative to all cells and is represented as mean ± SEM (n = 3). (D) CFSE-labeled CD14^+^ cells were added to 5 days old spheroids and co-cultured for 2 days. Infiltration was visualized via fluorescence microscopy.

MΦs have been shown to play an important role in supporting tumor progression and metastasis [14]. In order to explore how MФs influence tumor cells, we grew MCF7 breast tumor cells as three-dimensional tumor spheroids. After 5 days, the MCF7 tumor spheroids began to develop a characteristic necrotic core (Fig 1A) [15, 16], thus providing an *in vitro* proxy for the situation *in vivo*. Subsequently, CD14^+^ cells, i.e. monocytes, isolated from primary buffy coats were allowed to infiltrate into the spheroids [15, 16]. Flow cytometric analyses revealed that the CD14^+^ cells indeed infiltrated into the spheroids, resulting in 14.3 +/- 0.5% immune cells within the spheroid after 2 days of infiltration (Fig 1B and 1C). Infiltration into the spheroids was validated by labeling the CD14^+^ cells with carboxyfluorescein succinimidyl ester (CFSE) prior to infiltration. In line with the flow cytometric analyses, labeled cells were found within the tumor spheroids after 2 d of co-culture (Fig 1D).

**Fig 2:**
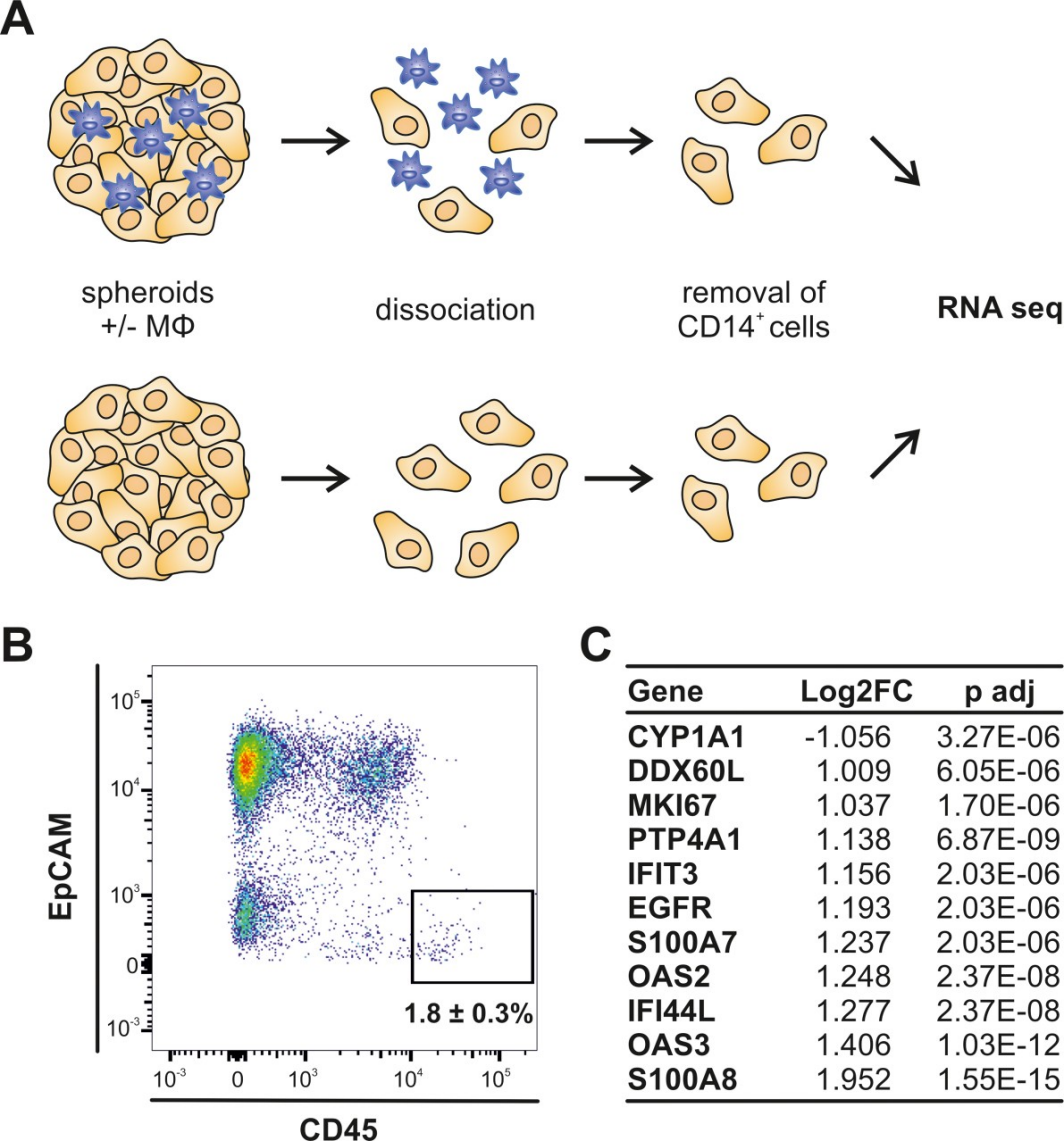
Tumor cell-specific gene expression changes after macrophage infiltration. (A) Schematic overview of the experimental setup of tumor cell isolation for RNA seq. (B) Purity of tumor cells after removal of CD14^+^ cells from dissociated tumor spheroids was determined by FACS analysis of tumor cells (EpCAM^+^) and immune cells (CD45^+^). Graph is representative of 3 independent experiments. The proportion of immune cells (CD45^+^) was quantified relative to all cells and is given as mean ± SEM (n = 3). (C) Top differentially expressed genes identified by RNA seq analysis of tumor cells from infiltrated relative to non-infiltrated MCF7 tumor spheroids.

To determine tumor cell specific transcriptional changes in response to MФ infiltration, CD14^+^ cells were separated from tumor cells prior to RNA extraction (Fig 2A). Purification was verified by FACS analysis underlining almost complete removal of infiltrated MФs from the tumor cells (Fig 2B). CD14^+^-depleted single cells from both infiltrated and non-infiltrated tumor spheroids were then analyzed by mRNA seq. While most mRNAs showed elevated expression in tumor cells upon MФ infiltration (Fig 2C), *CYP1A1* mRNA expression was down-regulated more than 2.08 fold (Log2FC = -1.06).

As contaminating mRNA from residual MФs might contribute to the false discovery of upregulated mRNAs, we selected *CYP1A1* for further investigations.

### Regulation of CYP1A1 mRNA expression by MФs

**Fig 3:**
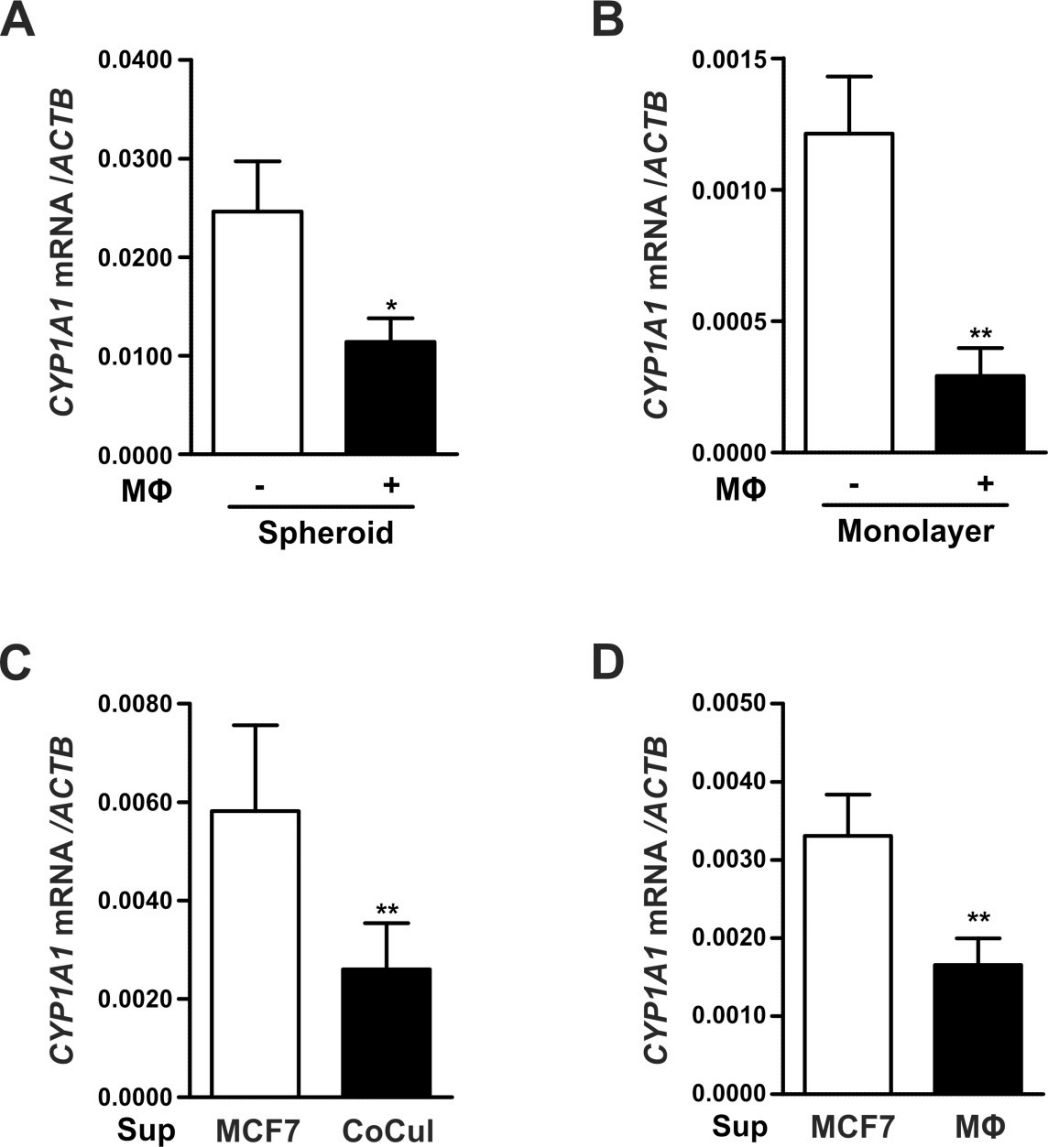
Macrophages suppress *CYP1A1* expression in breast tumor cells. (A) MCF7 cells grown as tumor spheroids were cultured for 48 hours in the absence or presence of CD14^+^ cells. (B) Monolayer MCF7 cells were co-cultured with MΦs. (C-D) Monolayer MCF7 cells were incubated with supernatants of MCF7 cells (Sup MCF7), (C) supernatants of MCF7-MΦ co-cultures (Sup CoCul), or (D) supernatants of MΦs alone (Sup MФ) for 48 hours. *CYP1A1* mRNA expression was determined by RT-qPCR analysis and normalized to *ACTB*. Data are presented as means ± SEM (n ≥ 3, * p < 0.05, ** p < 0.01).

Reduced *CYP1A1* mRNA expression (50%) in tumor spheroids after MФ infiltration was further verified using qPCR analyses (Fig 3A). Furthermore, *CYP1A1* mRNA expression was also reduced in tumor cells grown as monolayers after their co-culture with MФs (Fig 3B).

**Fig 4:**
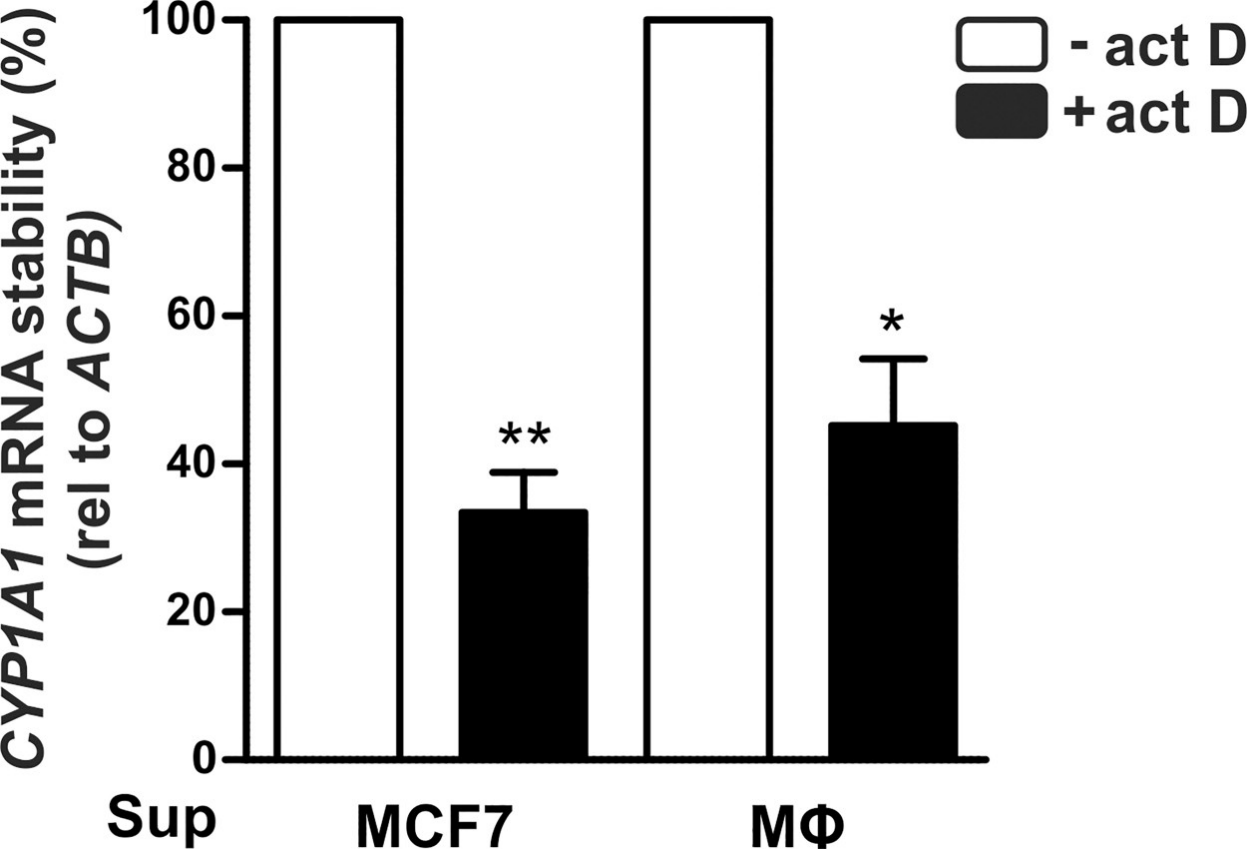
*CYP1A1* mRNA stability. MCF7 cells were incubated with supernatants of MCF7 cells or MΦs for 48 hours. *De novo* mRNA synthesis was blocked by addition of the transcription inhibitor actinomycin D (act D, 4 μg/ml) for the last 2 hours. *CYP1A1* mRNA expression was determined by RT-qPCR analysis and normalized to *ACTB*. mRNA stability is given as mean expression ± SEM after 2 h act D relative to cells incubated with the respective supernatants without addition of act D (n = 3, * p < 0.05, ** p < 0.01).

As a side note, *CYP1A1* was expressed at a higher basal level in tumor spheroids as compared to monolayer tumor cells, yet equally down-regulated by MΦs in both settings. To test if *CYP1A1* mRNA expression responded to elevated cell numbers rather than to a MФ-shaped environment, we analyzed *CYP1A1* expression in MCF7 cells grown under normal vs. high density conditions and observed no differences (S1 Fig). As these observations suggest that the *CYP1A1* expression changes are due to the MФ co-culture, we next aimed to determine if a direct cell-cell contact is required or if the regulation is facilitated via altered MФ-derived factors. Supernatants from MФs co-cultured with MCF7 cells, which display a tumor-associated MΦ (TAM)-like phenotype [17], inhibited *CYP1A1* expression as compared to supernatants of MCF7 cells (Fig 3C). Furthermore, supernatants from non-activated MФs alone sufficed to reduce *CYP1A1* expression in MCF7 cells (Fig 3D). Taken together, these data suggest that MФs, irrespective of their polarization or activation status, release factors which attenuate the expression of *CYP1A1* in the tumor cells. As *CYP1A1* mRNA expression has been reported to be regulated both transcriptionally and post-transcriptionally [18, 19], we decided to evaluate if MФ supernatants might regulate *CYP1A1* post-transcriptionally. To this end, we blocked transcription with actinomycin D for 2 hours to assess *CYP1A1* mRNA stability. We found that upon transcriptional blockade *CYP1A1* mRNA levels decreased similarly in MCF7 cells treated with supernatants of MCF7 cells as in those treated with supernatants of MФs (Fig 4).

Thus, MФs alter *CYP1A1* expression likely via transcriptional mechanisms. Yet, while *CYP1A1* transcription is commonly induced through the aryl hydrocarbon receptor (AhR), MФ supernatants were not able to inhibit AhR agonist 2,3,7,8-tetrachlorodibenzodioxin (TCDD)-induced expression of *CYP1A1* (S2 Fig), supporting an AhR-independent mode of transcriptional repression of *CYP1A1* in tumor cells elicited by MФs.

### Functional changes in tumor cells induced by MФs

**Fig 5:**
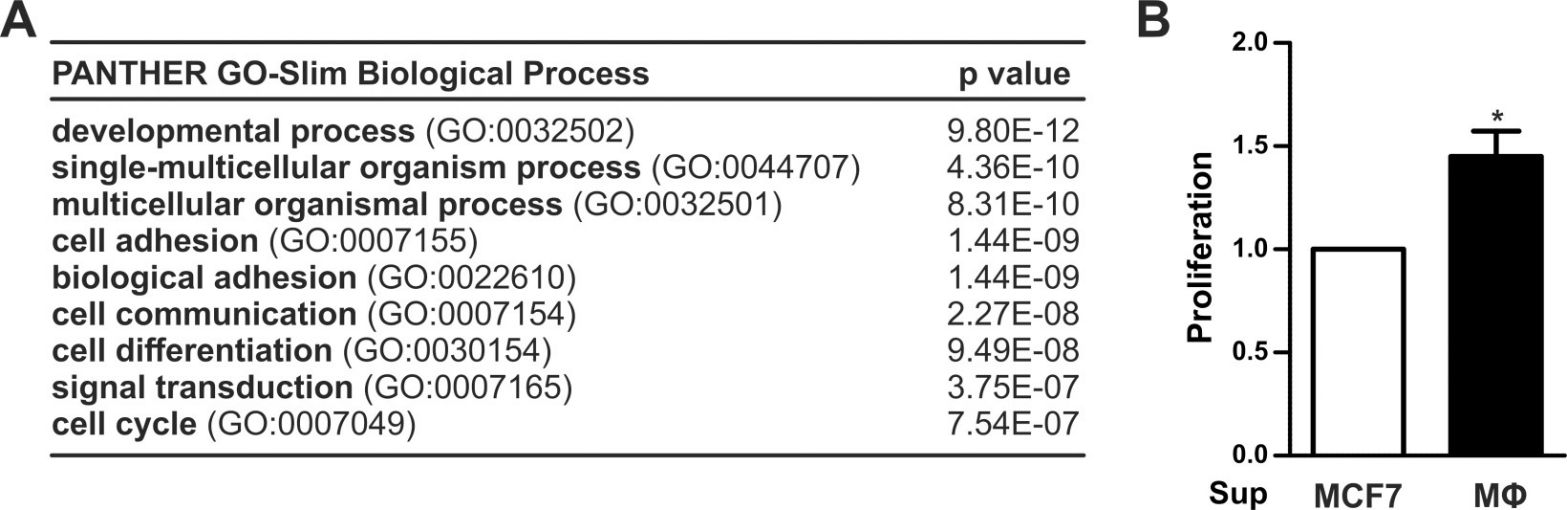
Functional impact of macrophages on breast tumor cells. (A) Enriched biological processes as determined by GO term analysis of the RNA seq data from MΦ-infiltrated and non-infiltrated tumor spheroids. (B) 1 x 10^4^ MCF7 cells were seeded in a 96-well plate and incubated with supernatants from MCF7 cells or MΦs. Proliferation was assessed using an IncuCyte S3 system and is presented as relative increase in confluency. Data are presented as means ± SEM (n > 3, * p < 0.05).

Next, we aimed to address functional consequences associated with MФ-tumor cell interactions. GO term analysis of the RNA seq data obtained for MФ-infiltrated as compared to non-infiltrated tumor spheroids yielded a number of tumor-associated processes positively affected by MФs, including cell cycle and adhesion (Fig 5A). We therefore tested the impact of *CYP1A1* repressive supernatants of non-activated MΦ on MCF7 cell proliferation. Indeed, MCF7 cells showed a significantly higher proliferation when exposed to MФ supernatants as compared to MCF7 supernatants (Fig 5B). In line, the RNA seq analysis showed a 2.05 fold (log2FC = 1.04) higher expression of the proliferation marker *MKI67* in tumor spheroids upon infiltration with MФs (Fig 2C).

**Fig 6:**
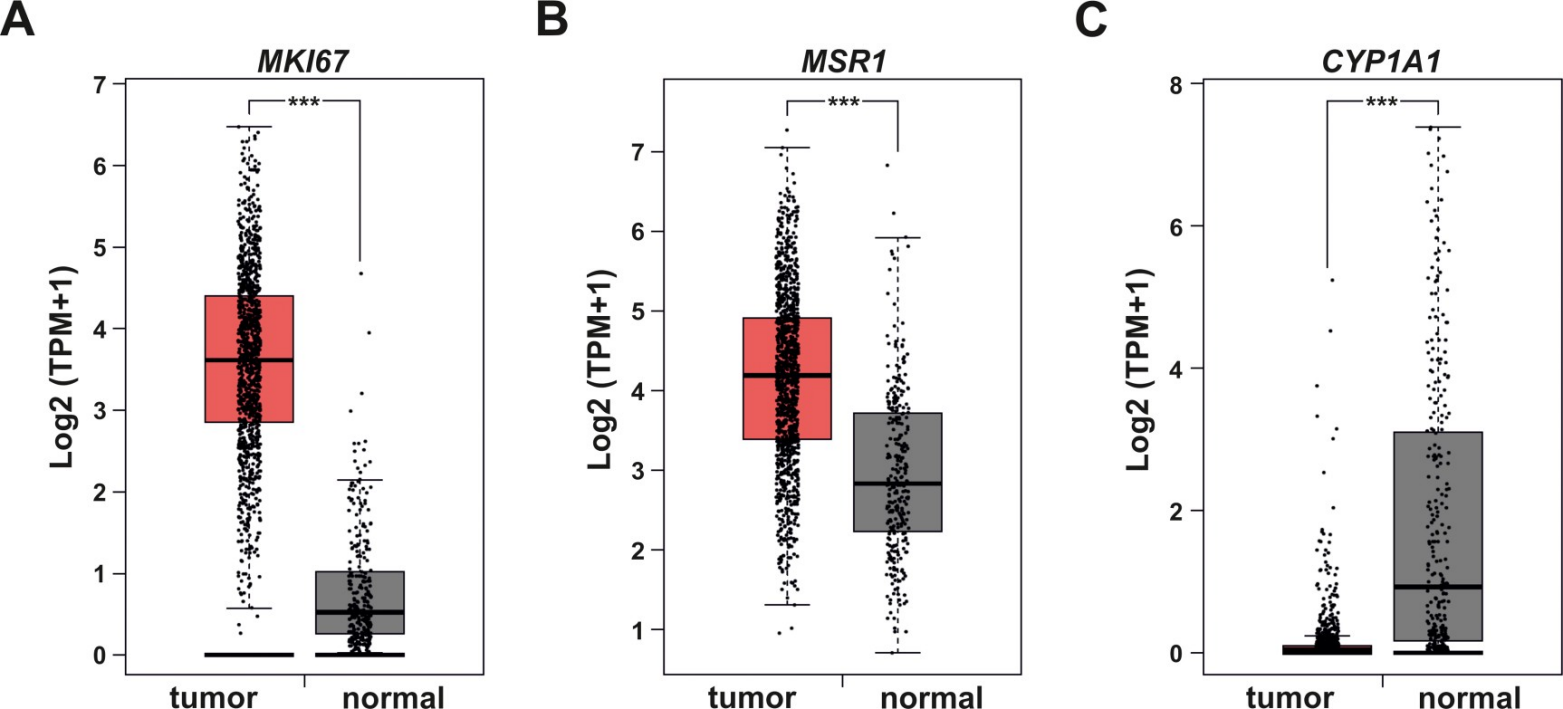
Clinical breast cancer data. For the evaluation of patient data publicly available breast cancer RNA seq data sets were analyzed using the interactive web server GEPIA. Expression of the proliferation marker *MKI67* (A), the MΦ marker *MSR1* (B), and *CYP1A1* (C) was compared between normal and tumor samples and is given as log2(TPM+1) (*** p < 0.001).

To evaluate if these findings translate into a clinical context, we compared mRNA expression in tumor vs. normal tissue samples out of publicly available databases (TCGA, GTEx). In line with our *in vitro* data, tumor samples displayed higher expression of the proliferation marker *MKI67* (Fig 6A) and also contained more MΦ marker *MSR1* (Fig 6B). In addition, *CYP1A1* was significantly lower in tumors as compared to normal tissue specimen (Fig 6C).

Taken together, the observation that MΦs repress *CYP1A1* expression in tumor cells and enhance their proliferation, nicely corroborates clinical data.

## Discussion

In the present study, we characterized the impact of MΦ-derived factors on breast tumor cells. We found that MΦs enhance the proliferation of MCF7 cells, which was paralleled by higher MΦ numbers in and enhanced proliferation of breast tumors vs. normal tissue. Moreover, MΦs markedly repressed the expression of the cytochrome P450 enzyme *CYP1A1*, which was also strongly reduced in primary tumors.

While earlier detection and additional therapeutic options have led to a better prognosis for breast cancer patients, the death toll due to breast cancer remains high [20]. Thus, it is crucial to further investigate molecular changes occurring in breast tumor cells to allow for the development of novel, targeted therapies. The development and progression of tumors is strongly influenced by the surrounding and infiltrating stroma [21, 22]. Thus, the characterization of changes in tumor cells occurring within the tumor microenvironment appears of the utmost importance. Within the tumor microenvironment, immune cells play a decisive role, and it has been appreciated that MΦs are present at high numbers in breast tumors and their presence constitutes a negative prognostic marker [3]. In line, we observed that a MΦ-shaped microenvironment increases the proliferation of MCF7 breast tumor cells, which was also supported by the positive enrichment of cell cycle-associated mRNAs (Fig 5). Furthermore, the elevated presence of MΦs in tumor vs. normal tissue of breast cancer patients was also paralleled by enhanced proliferation (Fig 6). Importantly, the elevated presence of MΦs in the tumor samples, needs to be taken into account when interpreting the expression data available in public data bases as these do not reflect tumor cell specific responses only.

As we aimed at characterizing the specific effects of MΦs on breast tumor gene expression, we used a three-dimensional tumor spheroid model, which is known to resemble the tumor situation *in vivo* more closely than a mere monolayer setting [23]. Such a model is further needed to allow for the infiltration and differentiation of MΦs similar to the situation *in situ*. Even though we used a simplified approach exclusively focusing on the interaction of tumor cells and MΦs, GO term analyses revealed that multicellular organismal processes where enriched in the RNA seq data of the tumor cells (Fig 5). With respect to molecular changes, we observed that while the expression of the majority of mRNAs increased in the tumor cells upon MΦ infiltration, *CYP1A1* expression appeared to be inhibited. This corroborates previous findings that inflammatory conditions, induced by LPS or found during mastitis, suppress *CYP1A1* expression in mammary epithelial cells [13]. In contrast to epithelial cells, enhanced *CYP1A1* expression in response to LPS was shown in human dendritic cells [24]. Similarly, the MΦs displaying a TAM-like phenotype upon co-culture with MCF7 cells had markedly elevated *CYP1A1* expression as compared to naïve MΦ (S3 Fig). These apparently contradictory observations indicate that the regulation of *CYP1A1* is highly cell type specific. The fact that *CYP1A1* is almost completely depleted in primary breast tumor specimen (Fig 6), suggests that a potentially increased expression of *CYP1A1* in the heterogeneous immune cell compartment under such tumor-associated inflammatory conditions does not suffice to compensate for the downregulation in the tumor cells. This might be of importance considering that the availability of cytochrome P450 enzymes in tumors can be predicted to affect the inactivation of therapeutic drugs as well as the toxification of certain compounds. Along these lines, we previously showed that *CYP1A1* expression was reduced in HIF-1α-depleted MΦs, which reduced biotransformation of the carcinogenic PAHs 7,12-dimethylbenz[a]anthracene (DMBA) and MCA into their DNA-damaging reactive intermediates. Consequently, reduced *CYP1A1* expression in mice with a myeloid HIF-1α knockout developed fewer tumors in the MCA-induced fibrosarcoma model [9]. In contrast, we observed that reduced *CYP1A1* expression in breast tumor cells, as observed upon contact with MΦs, correlates with enhanced proliferation of the respective tumor cells (Fig 5), nicely reflecting the situation found in patient samples (Fig 6). These differences might reflect different roles of *CYP1A1* during the initial transformation of cells, which commonly requires biotransformation activities, as compared to later stages of tumor promotion and progression, which are considered to be influenced by infiltrating immune cells. Interestingly though, *CYP1A1* repression in tumor cells induced by MΦ appeared to be independent of the exact MΦ phenotype. In fact, repression occurred in the complex 3D tumor spheroid model, in which monocytes are infiltrating and differentiating to MΦs, yet neither adopt a typical classical or alternative phenotype [15]. Similarly, *CYP1A1* was attenuated in a monolayer co-culture model, where fully differentiated MΦ were previously shown to take on a TAM-like phenotype [17], and even in response to supernatants of non-activated MΦs (Fig 3). Based on these findings, it is difficult to predict responsible MΦ generated factors. In fact, it might be envisioned that various factors might be relevant under different circumstances.

The role of CYP1A1 in tumor development appears to depend both on the cell type expressing *CYP1A1* as well as on the exact conditions. Since CYP1A1 acts not only as an activator of carcinogens, but also can activate certain pro-drugs [25], a detailed understanding of its expression within the tumor microenvironment appears of great interest. Appreciating the role of the immune response in tumor development, recent tumor therapeutic strategies target the tumor cells, at the same time aiming to direct the immune response against the tumors. In this context, our finding that *CYP1A1* expression is suppressed by MΦs should be considered *e*.*g*. for the appropriate choice of chemotherapeutic as some chemotherapeutics rely on the activation by CYP1A1 [26].

## Materials and methods

### Materials

All chemicals were purchased from Sigma-Aldrich, if not indicated otherwise.

### Cell culture

Human MCF7 breast cancer cells were purchased from ATCC-LGC GmbH and maintained in RPMI 1640 medium (Thermo Fisher Scientific) supplemented with 10% fetal bovine serum (Capricorn Scientific), 1% sodium pyruvate (Sigma-Aldrich), 100 U/ml penicillin, and 100 μg/ml streptomycin (Sigma-Aldrich). Cells were cultivated at 37°C in a humidified atmosphere with 5% CO_2_. Multicellular spheroids were generated according to the liquid overlay technique by seeding 7.5 x 10^3^ MCF7 cells per well in agarose-coated 96-well plates. Cells were subjected to centrifugation at 500 x g for 4 minutes and incubated for 5 days to obtain three-dimensional spheroid architecture.

### Primary MФ isolation and co-culture

Peripheral blood mononuclear cells (PBMCs) were prepared from human buffy coats (DRK-Blutspendedienst Baden-Württemberg-Hessen, Frankfurt, Germany) using Bicoll Separating Solution (Biochrom). Subsequently, CD14^+^ cells, i.e. monocytes, were isolated by magnetic cell sorting using microbeads for human CD14 (Miltenyi Biotec). For spheroid co-cultures, 5 days old spheroids were cultured with 7.5 x 10^4^ CD14^+^ cells for additional 2 days to allow for infiltration. To further validate the infiltration, CD14^+^ cells were labeled for 10 min with CFSE (eBioscience) prior to addition to the spheroids and evaluated fluorescence microscopically at the end of the infiltration.

For monolayer co-cultures, isolated PBMCs were seeded at a density of 80% in 15 cm dishes in RPMI 1640 medium including 100 U/ml penicillin and 100 μg/ml streptomycin. After adhesion, medium was changed to the same medium including 5% AB-positive human serum (DRK-Blutspendedienst Baden-Württemberg-Hessen, Frankfurt, Germany) to allow for differentiation of the adherent monocytes to MФs. Medium was changed every 2–3 days for 7 days, after which 4 x 10^6^ MCF7 cells were added for co-culture for 48 hours in MCF7 medium. Conditioned media of the MФs, the MΦ-MCF7 co-cultures, or pure MCF7 cells were collected after 48 hours, centrifuged at 1000 x g for 5 minutes at 4°C and stored at -80°C until further use.

### Tumor cell isolation after co-culture

Spheroids were washed with PBS, treated with Accutase (Sigma-Aldrich) for 20 minutes at 37°C and subjected to a cell strainer (35 μm, Corning) to obtain single cell suspensions. Cells were incubated with microbeads for human CD14 (Miltenyi Biotec) and tumor cells were isolated by negative selection after magnetic cell sorting. Monolayer cells were washed with PBS prior to dissociation of tumor cells through trypsinization for 5 minutes.

### Flow cytometry

Tumor cell spheroids before and after MФ depletion were washed with PBS and blocked with 4% FcR Blocking Reagent (Miltenyi Biotec) prior to antibody staining using EpCAM (CD326, Brilliant Violet421, BioLegend) and CD45 (Alexa Fluor 700, BioLegend). Cells were analyzed with a LSRII/Fortessa flow cytometer (BD Biosciences) and analyzed using FlowJo V10.

### RNA extraction and quantitative PCR

Total RNA was isolated using PeqGold RNAPure kit (PeqLab Biotechnology) and 1 μg RNA was reverse transcribed using the Maxima first strand cDNA synthesis kit (Thermo Fisher Scientific). Individual mRNAs were analyzed using the iQ SYBR Green Supermix on an CFX Connect and evaluated using the Bio-Rad CFX Manager (version 3.1) (all BioRad). Actin B served as internal control. The following primers (biomers.net) were used to detect the specific targets: *CYP1A1* (fwd: CTA CCC AAC CCT TCC CTG AAT; rev: CGC CCC TTG GGG ATG TAA AA), *ACTB* (fwd: ACC AAC TGG GAC GAC ATG GAG AAA; rev: TAG CAC AGC CTG GAT AGC AAC GTA). mRNA stability was assessed by comparing *CYP1A1* mRNA expression after 2 h transcriptional inhibition with actinomycin D (4 μg/ml) relative to the expression in the absence of actinomycin D.

### Cell proliferation assay

Proliferation of MCF7 cells in response to supernatants from MФs (Sup MФ) or MCF7 cells (Sup MCF7) was monitored for 24 hours using the IncuCyte S3 live cell imaging system (Essen BioScience). Cells were plated at a density of 1 x 10^4^ cells/well in a 96-well plate and changes in confluency were monitored for 4 hours after addition of the supernatants. Changes in the proliferation rate were calculated as increase in confluency in MCF7 cells exposed to MΦ supernatants relative to those in response to MCF7 supernatants.

### RNA seq

For sequencing, total RNA was extracted from MCF7 tumor cells isolated from 1000 spheroids infiltrated with MФs or not. Briefly, RNA was isolated out of 100 μl MCF7 tumor cell lysates using the RNA Clean and Concentrator-25 kit (Zymo Research). rRNA was removed using the RiboZero Gold rRNA Removal kit (Human/Mouse/Rat, Illumina). After heat fragmentation, end repair, and ligation of 3’ adapters, the RNA was reverse transcribed. The resulting cDNA was purified on 10% TBA polyacrylamide gels. Circularization, PCR amplification, and purification using Agencourt AMPure XP beads (Beckman Coulter), yielded sequencing ready libraries. The libraries were sequenced (single-end, 51 cycles) on a NextSeq500 sequencer (Illumina). Sequencing data were analyzed using the SeqBox software [27]. Briefly, after adapter trimming with skewer [28], reads were mapped to the human reference genome (hg38) using STAR [29]. Gene level quantification by RSEM [30] preceded the differential expression analysis by DESeq2 [31]. Enriched biological processes were identified using the Panther classification system (PANTHER version 13.1 Released 2018-02-03) [32]. NGS data have been deposited under the GEO accession number GSE119147.

### Clinical data

Expression of *CYP1A1*, *MSR1*, and *MKI67* was compared in RNA seq data of 1085 breast tumor samples and 291 normal breast samples available through The Cancer Genome Atlas (TCGA) and Genotype Tissue Expression (GTEx) projects using GEPIA [33]. Comparison was based on a log2FC cut-off of 0.5.

### Statistical analyses

Data are reported as mean ± SEM of at least three independent experiments and analyzed using two-tailed t-test in Prism software (GraphPadSoftware Inc.) unless otherwise stated.

## Supporting information

S1 Fig Effect of confluency on *CYP1A1* mRNA expression. MCF7 cells were grown at normal (4 x 10^6^ cells) or high density (8 x 10^6^ cells) in 15 cm dishes for 48 hours. *CYP1A1* mRNA expression was determined by RT-qPCR analyses and normalized to *ACTB*. Data are presented as means ± SEM (n = 3).
(DOCX)

S2 Fig *CYP1A1* induction by TCDD. MCF7 cells were treated for 2 hours with supernatants from MCF7 (Sup MCF7) or MΦs (Sup MФ), prior to stimulation with the AhR inducer 2,3,7,8-tetrachlorodibenzodioxin (TCDD, 30 μM, Sigma-Aldrich) or vehicle control (Ctrl) for additional 4 hours. *CYP1A1* mRNA expression was determined by RT-qPCR analyses and normalized to *ACTB*. Data are presented as means ± SEM (n = 3, ** p < 0.01).
(DOCX)

S3 Fig *CYP1A1* expression in macrophages. Macrophages were cultured alone or co-cultured with MCF7 cells for 48 h. Before harvesting the macrophages, tumor cells were removed by trypsinization. *CYP1A1* mRNA expression was determined by RT-qPCR analyses and normalized to *ACTB*. Data are presented relative to macrophages only as means ± SEM (n = 3, * p < 0.05).
(DOCX)
