# Supplementary material for: Macrophages attenuate the transcription of CYP1A1 in breast tumor cells and enhance their proliferation
Source: PLoS One. 2019 Jan 7;14(1):e0209694. doi: 10.1371/journal.pone.0209694 (PMC6322746; doi:10.1371/journal.pone.0209694)
Supplement: S1 Fig — MCF7 cells were grown at normal (4 x 10^6^ cells) or high density (8 x 10^6^ cells) in 15 cm dishes for 48 hours. CYP1A1 mRNA expression was determined by RT-qPCR analyses and normalized to ACTB. Data are presented as means ± SEM (n = 3). (DOCX) [file pone.0209694.s001.docx]

**S1 Fig.** **Effect of confluency on *CYP1A1* mRNA expression.** MCF7 cells were grown at normal (4 x 10^6^ cells) or high density (8 x 10^6^ cells) in 15 cm dishes for 48 hours. *CYP1A1* mRNA expression was determined by RT-qPCR analyses and normalized to *ACTB*. Data are presented as means ± SEM (n=3).
