# Supplementary material for: Macrophages attenuate the transcription of CYP1A1 in breast tumor cells and enhance their proliferation
Source: PLoS One. 2019 Jan 7;14(1):e0209694. doi: 10.1371/journal.pone.0209694 (PMC6322746; doi:10.1371/journal.pone.0209694)
Supplement: S2 Fig — MCF7 cells were treated for 2 hours with supernatants from MCF7 (Sup MCF7) or MΦs (Sup MФ), prior to stimulation with the AhR inducer 2,3,7,8-tetrachlorodibenzodioxin (TCDD, 30 μM, Sigma-Aldrich) or vehicle control (Ctrl) for additional 4 hours. CYP1A1 mRNA expression was determined by RT-qPCR analyses and normalized to ACTB. Data are presented as means ± SEM (n = 3, ** p < 0.01). (DOCX) [file pone.0209694.s002.docx]

**S2 Fig. *CYP1A1* induction by TCDD.** MCF7 cells were treated for 2 hours with supernatants from MCF7 (Sup MCF7) or MΦs (Sup MФ), prior to stimulation with the AhR inducer 2,3,7,8-tetrachlorodibenzodioxin (TCDD, 30 µM, Sigma-Aldrich) or vehicle control (Ctrl) for additional 4 hours. *CYP1A1* mRNA expression was determined by RT-qPCR analyses and normalized to *ACTB*. Data are presented as means ± SEM (n=3, ** p < 0.01).
