# Supplementary material for: Macrophages attenuate the transcription of CYP1A1 in breast tumor cells and enhance their proliferation
Source: PLoS One. 2019 Jan 7;14(1):e0209694. doi: 10.1371/journal.pone.0209694 (PMC6322746; doi:10.1371/journal.pone.0209694)
Supplement: S3 Fig — Macrophages were cultured alone or co-cultured with MCF7 cells for 48 h. Before harvesting the macrophages, tumor cells were removed by trypsinization. CYP1A1 mRNA expression was determined by RT-qPCR analyses and normalized to ACTB. Data are presented relative to macrophages only as means ± SEM (n = 3, * p < 0.05). (DOCX) [file pone.0209694.s003.docx]

**S3 Fig. *CYP1A1* expression in macrophages.** Macrophages were cultured alone or co-cultured with MCF7 cells for 48 h. Before harvesting the macrophages, tumor cells were removed by trypsinization. *CYP1A1* mRNA expression was determined by RT-qPCR analyses and normalized to *ACTB*. Data are presented relative to macrophages only as means ± SEM (n=3, * p < 0.05).
